# Prevention of Work-Related Musculoskeletal Disorders

**DOI:** 10.1186/2052-4374-26-14

**Published:** 2014-06-24

**Authors:** Dongmug Kang, Young-Ki Kim, Eun-A Kim, Dae Hwan Kim, Inah Kim, Hyoung-Ryoul Kim, Kyoung-Bok Min, Kyunghee Jung-Choi, Sung-Soo Oh, Sang-Baek Koh

**Affiliations:** 1 Department of Occupational and Environmental Medicine, Pusan National University, Yangsan Hospital, Yangsan, Korea; 2 Department of Preventive and Occupational Medicine, Pusan National University, School of Medicine, Yangsan, Gyongnam, Korea; 3 Occupational Safety & Health Research Institute, Korea Occupational Safety & Health Agency, Ulsan, Korea; 4 Department of Occupational and Environmental Medicine, Haeundae Paik Hospital, Inje University, Busan, Korea; 5 Department of Occupational and Environmental Health, Yonsei University Graduate School of Public Health, Seoul, Korea; 6 Department of Occupational and Environmental Medicine, College of Medicine, the Catholic University, Seoul, Korea; 7 Department of Occupational and Environmental Medicine, Ajou University School of Medicine, Suwon, Korea; 8 Department of Preventive Medicine, Ewha Womans University School of Medicine, Seoul, Korea; 9 Department of Occupational and Environmental Medicine, Wonju Severance Christian Hospital, Wonju College of Medicine, Yonsei University, Wonju, Korea; 10 Department of Preventive Medicine and Institute of Occupational and Environmental Medicine, Wonju College of Medicine, Yonsei University, Wonju, Korea

## Editorial

Work-related musculoskeletal disorders (WMSDs) constitute a major component of occupational diseases (ODs), accounting for approximately 38.1% of all ODs in Europe [1] and approximately 70% of all compensated ODs in Korea [2]. According to a recent European Union report, WMSDs tend to be underreported and are tending to increase among women, young, and migrant workers. The costs for upper extremity WMSDs alone rage from 0.5 to 3.8% of gross national product [3]. In the United States, costs for compensation, wage loss, and production loss range from 45–54 billion US dollors [4]. Research to prevent these highly costly WMSDs is being conducted in various fields including epidemiology, physiology, ergonomics, biomechanics, molegular biology, and genetics and to tackle such issues as return to work, rehabilitation, policy and compensation. One of the most important avenues of communication for these research efforts is the Intenational Conference on Prevention of Work-related Musculoskeletal Disorders (PREMUS).

There are 35 active scientific committees in the International Commission on Occupational Health, and the Musculoskeletal Disorder Committee has held an international conference every 3 years under the name of PREMUS. The most recent PREMUS conference was held in Busan, Korea on July 7–11, 2013. It was the first time that PREMUS was organized outside of North America or Europe. Attending pariticipants were 290 scholars from 30 conutries all over the world, including 11 Asian countries. Among the many papers presented at the conference, 10 describing the spectrum of WMSDs research around world were chosen to public in AOEM.

The study by Nur Azmar et al. shows the prevalence and psychosocial risk factors for WMSDs among Malaysian nurses. Another study by Shimaoka et al. deals with workload problems in Japanese health personnel. In another report, Sato et al. demonstrate the relationship between psychosocial indicators and WMSDs among Brazilian workers. Mohandoss et al. describe the prevalence of WMSDs and comorbidity with neck pain among information technology (IT) professional in India, whereas Sharan et al. focus on IT device users. Kang et al. present the worker’s struggle against workload increase, specifically work intensification as a result of neoliberalized globalization. Kim and Nakata illustrate the similarity and differences between Korea and Japan within the context of the social system, which calls for international comparison studies. Gangopadhyay and Dev consider the feasibility of low-cost ergonomic intervention in developing countries including India. Kuijer et al. suggest multidisciplinary practice guidelines for lifting based on evidence. Spector et al. demonstrate a new automation technique to correct the NIOSH lifting equation to prevent back problems.

In this special issues, 10 papers by authors from developing to developed countries, subjects from different working population, and research methods from descriptive to intervention studies, are presented.

Greater efforts including international collaborative research for standardization and unification of disease definition and study methodology along with extension of insight combining micro and macro aspects are needed.
